# Antidepressant treatment and mortality in people with comorbid depression and type 2 diabetes: UK electronic health record study

**DOI:** 10.1192/bjo.2024.33

**Published:** 2024-04-12

**Authors:** Annie Jeffery, Kate Walters, Ian C. K. Wong, David Osborn, Joseph F. Hayes

**Affiliations:** Epidemiology and Applied Clinical Research Department, Division of Psychiatry, University College London, UK; Department of Primary Care & Population Health, Institute of Epidemiology & Health Care, University College London, UK; Research Department of Practice and Policy, School of Pharmacy, University College London, UK; and Centre for Safe Medication Practice and Research, Department of Pharmacology and Pharmacy, Li Ka Shing Faculty of Medicine, The University of Hong Kong, Hong Kong

**Keywords:** Antidepressants, mortality, primary care, depressive disorders, type 2 diabetes

## Abstract

**Background:**

Depression is associated with higher rates of premature mortality in people with physical comorbidities, such as type 2 diabetes. Conceptually, the successful treatment of depression in people with type 2 diabetes could prevent premature mortality.

**Aims:**

To investigate the association between antidepressant prescribing and the rates of all-cause and cause-specific (endocrine, cardiovascular, respiratory, cancer, unnatural) mortality in individuals with comorbid depression and type 2 diabetes.

**Method:**

Using UK primary care records between years 2000 and 2018, we completed a nested case–control study in a cohort of people with comorbid depression and type 2 diabetes who were starting oral antidiabetic treatment for the first time. We used incident density sampling to identify cases who died and matched controls who remained alive after the same number of days observation. We estimated incidence rate ratios for the association between antidepressant prescribing and mortality, adjusting for demographic characteristics, comorbidities, medication use and health behaviours.

**Results:**

We included 5222 cases with a recorded date of death, and 18 675 controls, observed for a median of 7 years. Increased rates of all-cause mortality were associated with any antidepressant prescribing during the observation period (incidence rate ratio 2.77, 95% CI 2.48–3.10). These results were consistent across all causes of mortality that we investigated.

**Conclusions:**

Antidepressant prescribing was highly associated with higher rates of mortality. However, we suspect that this is not a direct causal effect, but that antidepressant treatment is a marker of more severe and unsuccessfully treated depression.

Depression is the leading cause of disability worldwide,^1^ partly because of its effects on physical health. People with type 2 diabetes are up to three times more likely to suffer from depression than people without type 2 diabetes.^2^ When the conditions are comorbid, depression is associated with poor glycaemic control,^3^ decreased adherence to diabetic treatments^4^ and the development of serious diabetic complications.^5^ In addition, depression may increase the risk of premature mortality by up to 1.5 times for people with type 2 diabetes.^6^ As such, the successful treatment of depression in people with type 2 diabetes is important for improving both physical and mental health outcomes. Conceptually, this may reduce preventable mortality in this patient group.

For individuals with type 2 diabetes, there is evidence that antidepressant treatment is effective in improving depressive symptoms and glycaemic control in the short term.^7^ However, antidepressants may also cause side-effects such as weight gain, nausea and cardiac disturbances,^8,9^ which may exacerbate type 2 diabetes and its complications, or interact with side-effects from antidiabetic medication. There is a lack of evidence concerning the long-term effects of antidepressant treatment on physical health outcomes in people with type 2 diabetes.^10^ Thus, although there is a need to treat depression in this patient group, the long-term effects of antidepressant treatment on physical health, and ultimately mortality, are unclear. We are aware of only one study investigating the association between antidepressant treatment and mortality in people with comorbid depression and diabetes (type unspecified): a large (*N* = 53 412) population-based cohort study in Taiwan, which found that people with comorbid depression and diabetes who were prescribed antidepressants had reduced rates of mortality compared with those who were not prescribed antidepressants.^11^ However, this study only measured whether an antidepressant was prescribed at the start of the study follow-up, which may have been ≥10 years before the outcome occurred. Because of the episodic nature of depression, antidepressant prescribing can also vary greatly over time, and so may be subject to change during this period. The study also did not adjust for other physical comorbidities, which may confound the relationship between antidepressant prescribing and mortality, as there is evidence that physical comorbidities, which may cause mortality, are associated with antidepressant prescribing.^12^ Finally, the study did not distinguish between different causes of mortality. This may obscure harmful effects leading to one cause (as a result of adverse side-effects) behind protective effects from another (if antidepressants reduce depressive symptoms, which then reduces mortality from associated causes of death).

## Study aims

We aimed to investigate the association between antidepressant prescribing and both all-cause and cause-specific mortality in adults with comorbid depression and type 2 diabetes, accounting for the time-varying nature of antidepressant prescribing and adjusting for the potentially confounding effects of other comorbidities. We hypothesised that antidepressant prescribing would be associated with decreased rates of mortality based on the assumption that successfully treating depression would improve physical health outcomes by improving mental health, and based on the short-term evidence that antidepressants improve glycaemic control. Second, we aimed to investigate differences in mortality rates according to the timing of the antidepressant prescribing. We hypothesised that we would see no difference in mortality rates according to the timing of the antidepressant, based on the assumption that antidepressant medication would not cause immediate harm resulting in mortality. Third, we aimed to investigate the association between the cumulative duration of antidepressant prescribing and mortality. We hypothesised that we would see no difference in mortality rates according to the duration of antidepressant treatment. This was based on the assumption that antidepressants would not cause harm (which would have a dose–response effect on mortality); rather, they would reduce mortality through the successful treatment of depression (which would have a binary, rather than dose–response relationship). Finally, we aimed to investigate differences in mortality rates according to the number of different antidepressant agents an individual was prescribed. The prescription of multiple different antidepressant agents may suggest that depression has been ‘complex to treat’. Therefore, we hypothesised that individuals who were prescribed the highest number of different antidepressant agents would represent those for whom depression was not being successfully treated, and so we would no longer see a decrease in mortality rates for these individuals.

## Method

The authors assert that all procedures contributing to this work comply with the ethical standards of the relevant national and institutional committees on human experimentation and with the Helsinki Declaration of 1975, as revised in 2008. All procedures involving human patients were approved by the Independent Scientific Advisory Committee of the Clinical Practice Research Datalink (CPRD) (protocol number 21_001648). All data sent to the CPRD is anonymised, and therefore consent is not required.

### Setting and design

We used longitudinal data from the CPRD, an electronic health record (EHR) data-set containing primary care records for over 60 million people, across 2000 practices in the UK.^13^ The CPRD includes two separate databases, Gold and Aurum, based on different computer software packages used for the EHRs, which we combined. The CPRD has been shown to be representative of the UK population with respect to age, gender and ethnicity.^14,15^ For eligible practices (these included practices in England only who had consented to data linkage), patient EHRs are linked to death registration data from the Office for National Statistics (ONS).^13^ We used the subset of CPRD data that is eligible for ONS mortality data linkage.

Our study period ran from 1 January 2000 to 31 December 2018. However, we used data from earlier years (pre-2000) to select the cohort and identify some of the confounder variables.

**Fig. 1:**
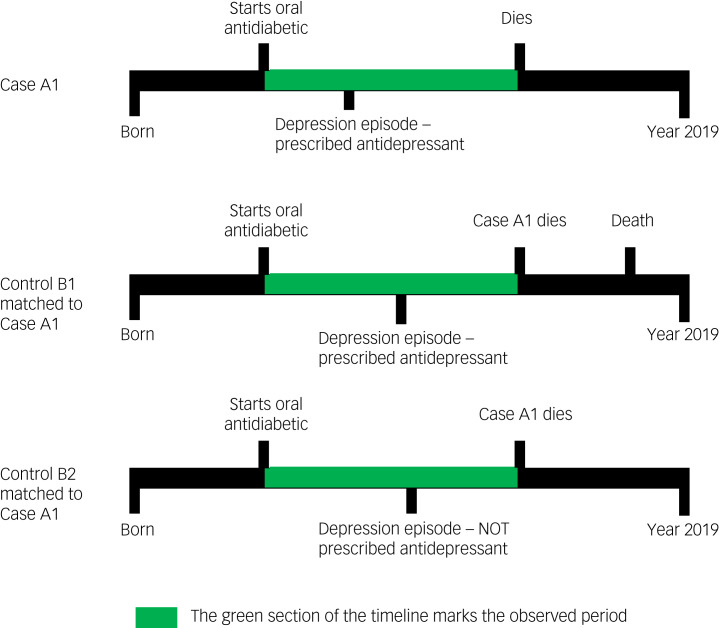
Examples of participant timelines during which exposure to antidepressant treatment was evaluated for both cases (who died) and controls (who did not die).

We used a nested case–control study design. A graphical representation of the study design is given in Fig. 1.

### Patient inclusion criteria

We nested our study within a cohort of adults (age ≥18 years) with comorbid depression and newly treated type 2 diabetes.

We identified individuals with depression as those who had any record of depressive symptoms, diagnoses or processes of care, after starting first-line treatment for type 2 diabetes. This included those who were newly depressed and those who had a previous history of depression. We excluded individuals who only had records of depression specific to dementia, maternity, schizophrenia or bipolar disorder.

We identified individuals with type 2 diabetes as those who had at least one oral antidiabetic prescription recorded during their EHR follow-up, with the first oral antidiabetic prescription dated at least 6 months after the individual's date of registration, to ensure we were capturing the start of treatment. As antidiabetic medication such as metformin may also be used to treat non-diabetic conditions,^16^ we also required that individuals had at least two blood/serum glucose/haemoglobin A1C tests recorded above the threshold for type 2 diabetes. We excluded individuals with <6 months between the date of the first recorded oral antidiabetic prescription and the first recorded insulin prescription (possible type 1 diabetes), or individuals who only had medication for type 2 diabetes prescribed during periods of pregnancy (possible gestational diabetes).

We defined the study entry date as the date of a participant's first oral antidiabetic prescription. We censored individuals at their date of death, end of registration with the general practice or end of the study period (31 December 2018), whichever was first.

### Selection of cases: individuals with a recorded date of death

We defined cases as individuals with a date of death recorded after their study entry date and before or on their censored date. We defined the outcome date as the date of death. We calculated the observation period as the number of days between starting oral antidiabetic medication (study entry date) and the date of death (outcome date). We excluded cases who could not be matched to one or more suitable controls.

### Selection of controls: individuals without a recorded date of death

We included all individuals from the cohort in which this case–control study was nested in the risk-set from which potential controls were selected, regardless of whether they later became a case.

We matched all cases to up to four randomly selected eligible controls. Eligible controls were participants who were included in the base cohort for at least as many days as the case, with a code for depression but no record of death during this time. Eligible controls were matches for a case based on the age at study entry (within 5 years), gender and GP practice.

We defined the outcome date for controls to be after same number of days as their matched case, with respect to the number of days observation period from study entry to the date of death. This ensured cases and controls had the same duration of observation period.

### Outcome subgroups for cause-specific mortality

We used linked ONS data to identify the primary cause of death, and categorised cases into the following subgroups: endocrine, cancer, cardiovascular, respiratory and any unnatural cause (including suicide). For the natural causes of death, the subgroups included were based on broad ICD-10 categories for the most common non-communicable natural causes of death. We excluded cases that did not fit into one of these categories from the cause-specific mortality analysis. Controls followed their respective cases into the different cause-specific mortality subgroups for analysis.

### Primary exposure of any antidepressant prescribing during the observation period

We defined the primary exposure as being prescribed one or more antidepressant between the study entry and the outcome date. We included antidepressant medications that were licensed for use in treating depression in the UK during the study period (see Supplementary Material available at https://doi.org/10.1192/bjo.2024.33).

### Secondary exposures: subcategories of antidepressant prescribing

We performed secondary analyses on three subcategories of the primary exposure (the reference category for each of these was no antidepressant prescription during the observed study period).

- Recent or past antidepressant use: We defined recent as any antidepressant prescription within 182 days (6 months) of the outcome date. We defined past as only having antidepressant prescriptions >182 days before the outcome date.
- Cumulative duration: We calculated an individual's cumulative duration of antidepressant treatment as the sum of the duration of treatment episode (the number of days between the first and last prescription of any antidepressant, plus the duration of that last prescription). If a participant had a gap of >60 days after the expected end date of the previous prescription without any subsequent antidepressant prescription recorded, that was considered to be the last prescription in that treatment episode. We counted any subsequent prescriptions as new treatment episodes, and included them in the total cumulative duration. We categorised the cumulative duration of antidepressant treatment into four groups, namely <6 months, 6–12 months, 13–24 months and >24 months.
- Number of different antidepressant agents prescribed during the observation period: We categorised these into four groups: 0 (reference category), 1, 2 and ≥3.

### Confounders

We included the following as potential confounders: the demographic factors used for matching (age, gender, GP practice), ethnicity, comorbidities, body mass index, smoking status, type 2 diabetes duration, number of primary care contacts, polypharmacy count and antidepressant prescribing history before study entry. Full details of confounding variables, including rationale for inclusion, are given in the Supplementary Material. We did not include glycaemic control as a potential confounder, as it is not well enough recorded in the data. However, given that participants entered the study when they first started antidiabetic medication, we would expect them to have uncontrolled blood sugar levels (which is the indication to start treatment) at this time.

### Statistical analysis

We used conditional logistic regression to estimate adjusted incidence rate ratios (IRRs) and corresponding 95% confidence intervals for the association between each of the antidepressant prescribing exposures and each mortality cause. Conditional logistic regression estimates IRRs in case–control studies where incident density sampling and individual matching is used.^17^ We initially performed univariable analyses, followed by then multivariable analyses adjusting for all aforementioned confounders.

### Sensitivity analyses

We performed two sensitivity analyses (for the outcome of all-cause mortality and primary exposure of any antidepressant prescribing) to investigate the role of imputing missing data. In the first, we included only individuals with complete data for ethnicity. In the second, we included only individuals with complete data for body mass index.

## Results

**Fig. 2:**
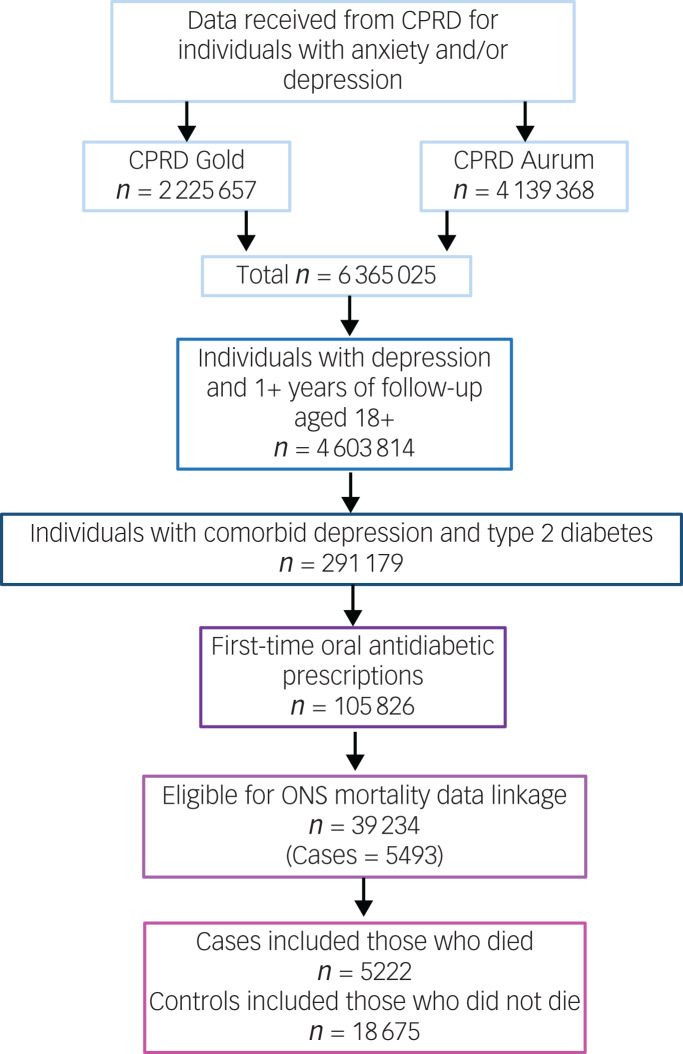
Flow diagram of inclusion and exclusion. CPRD, Clinical Practice Research Datalink; ONS, Office for National Statistics.

The number of individuals included or excluded is demonstrated in a flow diagram (Fig. 2)

The cohort in which this study was nested consisted of 39 234 individuals with comorbid depression and type 2 diabetes, who started oral antidiabetic medication during their EHR follow-up and were eligible for ONS mortality data linkage. From this, we identified 5222 cases who had a recorded date of death, and 18 675 controls who were presumed still alive at the time of their respective case's date of death. Individuals included in the study were observed for a median of 7.05 years (interquartile range 4.25–10.20).

Out of 5222 cases with comorbid depression and type 2 diabetes who died after starting oral antidiabetic medication, 207 (3.96%) died from endocrine causes, 1635 (31.31%) died from cardiovascular causes, 1474 (28.23%) died from cancer, 789 (15.11%) died from respiratory causes and 137 (2.62%) died from any unnatural cause (including suicide).

Table 1 contains the baseline characteristics of all individuals included in the study, and categorised as cases or controls. The median age of participants at the time of study entry was 69 years (interquartile range 61–76). Cases and controls were generally balanced in terms of baseline characteristics, with the exception of cases having higher rates of most comorbidities.

**Table 1:** Baseline characteristics of individuals included in the study

	All, *n* (%)	Cases, *n* (%)	Controls, *n* (%)
Sample size	23 897	5222	18 675
Female (%)	11 792 (49.35)	2579 (49.39)	9213 (49.33)
Median age (IQR) (years)	69 (61–76)	70 (62–77)	69 (60–75)
Ethnicity			
Asian (%)	908 (3.80)	136 (2.60)	772 (4.13)
Black (%)	345 (1.44)	54 (1.03)	291 (1.56)
Missing^a^ (%)	9032 (37.80)	2432 (46.57)	6600 (35.34)
Mixed (%)	58 (0.24)	7 (0.13)	51 (0.27)
Other (%)	72 (0.30)	4 (0.08)	68 (0.36)
White (%)	13 482 (56.42)	2589 (49.58)	10 893 (58.33)
Median type 2 diabetes duration in months (IQR)	6.11 (0.12–35.88)	6.78 (0.23–35.73)	5.91 (0.13–35.91)
Median (IQR) medication count (past 3 months)	6 (4–9)	6 (4–9)	6 (4–9)
Prescribed antidepressants in past 12 months	6640 (27.79)	1343 (25.72)	5297 (28.36)
Median primary care contacts in past 12 months	26 (15–40)	26 (15–40)	26 (16–40)
Median years observed (IQR)	7.05 (4.25–10.20)	7.05 (4.25–10.20)	7.05 (4.25–10.20)
Comorbidity			
Alcohol misuse	1399 (5.85)	382 (7.32)	1017 (5.45)
Blood loss anaemia	28 (0.12)	9 (0.17)	19 (0.10)
Cardiac arrhythmia	6225 (26.05)	1616 (30.95)	4609 (24.68)
Chronic heart failure	4031 (16.87)	1276 (24.44)	2755 (14.75)
Coagulopathy	496 (2.08)	144 (2.76)	352 (1.88)
COPD	9133 (38.22)	2208 (42.28)	6925 (37.08)
Deficiency anaemia (iron/B12)	4234 (17.72)	970 (18.58)	3264 (14.48)
Drug misuse	391 (1.64)	92 (1.76)	299 (1.60)
Fluid and electrolyte disorders	2883 (12.06)	771 (14.76)	2122 (11.36)
Hypertension (uncomplicated)	17 955 (75.13)	3794 (72.65)	14 161 (75.83)
Hypertension (late stage)	106 (0.44)	36 (0.69)	70 (0.37)
Hypothyroidism	3264 (13.66)	764 (14.63)	2500 (13.39)
Liver disease	900 (3.77)	281 (5.38)	619 (3.31)
Lymphoma (history)	338 (1.41)	110 (2.11)	228 (1.22)
Metastatic cancer	792 (3.31)	405 (7.76)	387 (2.07)
Other neurological disorders^b^	7942 (33.23)	2072 (39.68)	5870 (31.43)
Paralysis	294 (1.23)	99 (1.90)	195 (1.04)
Peptic ulcer disease	1830 (7.66)	469 (8.98)	1361 (7.29)
Peripheral vascular disease	2995 (12.53)	880 (16.85)	2115 (11.33)
Psychosis	920 (3.85)	252 (4.83)	668 (3.58)
Pulmonary circulation disorders	1172 (4.90)	374 (7.16)	798 (4.27)
Collagen vascular diseases	2403 (10.06)	550 (10.53)	1853 (9.92)
Renal disease	9285 (38.85)	2311 (44.26)	6974 (37.34)
Solid tumour or leukaemia	6788 (28.41)	1963 (37.59)	4825 (25.84)
Valvular disease	1841 (7.70)	456 (8.73)	1385 (7.42)
Smoking status			
Non-smoker	12 091 (50.60)	2702 (51.74)	9389 (50.28)
Current smoker	5413 (22.65)	1350 (25.85)	4063 (21.76)
Ex-smoker	6393 (26.75)	1170 (22.41)	5223 (27.97)
BMI category			
Missing^c^	8180 (34.23)	1960 (37.53)	6221 (33.31)
Normal	1633 (6.83)	418 (8.00)	1215 (6.51)
Overweight	4583 (39.76)	1862 (35.66)	7639 (40.90)
Obese	9501 (19.18)	983 (18.82)	3600 (19.28)

IQR, interquartile range; COPD, chronic obstructive pulmonary disease; BMI, body mass index.

a. Missing ethnicity imputed as ‘White’.

b. Other neurological disorders included brain trauma, cerebrovascular diseases, dementia, epilepsy, encephalitis, hydrocephalus, movement disorders, neurodegenerative diseases, other cerebral degeneration, spine injuries and disorders.

c. Missing data for BMI imputed using multiple imputation.

### Results for the association between antidepressant prescribing and mortality in individuals with comorbid depression and type 2 diabetes

Table 2 contains all results for the association between antidepressant prescribing and mortality. All of the results described narratively below are after adjustment for confounders.

**Table 2:** Incidence rate ratios and 95% confidence intervals for the association between antidepressant prescribing and mortality

Mortality cause	Exposure	Cases, *n* (%)	Controls, *n* (%)	Univariable IRR (95% CI)	Multivariable IRR (95% CI)
Any antidepressant versus no antidepressant					
All-cause	No antidepressant	1460 (27.96)	9884 (52.93)	Reference	Reference
	Any antidepressant	3762 (72.04)	8791 (47.07)	3.24 (3.02–3.49)	2.77 (2.48–3.10)
Endocrine	No antidepressant	52 (25.12)	387 (52.02)	Reference	Reference
	Any antidepressant	155 (74.88)	357 (47.98)	3.51 (2.42–5.09)	3.65 (1.83–7.29)
Cardiovascular	No antidepressant	483 (29.56)	3083 (53.03)	Reference	Reference
	Any antidepressant	1152 (70.46)	2791 (46.97)	2.93 (2.58–3.33)	2.27 (1.86–2.77)
Cancer	No antidepressant	445 (30.19)	2878 (53.11)	Reference	Reference
	Any antidepressant	1030 (69.88)	2546 (46.98)	2.98 (2.60–3.40)	2.94 (2.27–3.83)
Respiratory	No antidepressant	201 (25.48)	1515 (54.79)	Reference	Reference
	Any antidepressant	588 (74.52)	1293 (46.76)	3.82 (3.15–4.64)	2.94 (2.13–4.04)
All unnatural	No antidepressant	23 (16.79)	261 (52.41)	Reference	Reference
	Any antidepressant	114 (83.21)	237 (47.59)	7.28 (4.24–12.53)	9.27 (3.02–28.47)
Timing of the antidepressant prescription					
All-cause	No antidepressant	1460 (27.96)	9884 (52.93)	Reference	Reference
	Recent antidepressant	2742 (52.51)	6080 (32.56)	3.41 (3.16–3.68)	3.19 (2.82–3.61)
	Past antidepressant	1020 (27.11)	2711 (14.52)	2.85 (2.59–3.14)	2.14 (1.84–2.48)
Endocrine	No antidepressant	52 (25.12)	387 (52.02)	Reference	Reference
	Recent antidepressant	245 (32.93)	820 (50.18)	3.78 (2.56–5.57)	4.45 (2.10–9.43)
	Past antidepressant	112 (15.05)	332 (20.32)	2.84 (1.69–4.66)	2.08 (0.80–5.37)
Cardiovascular	No antidepressant	483 (29.56)	3083 (53.03)	Reference	Reference
	Recent antidepressant	820 (50.18)	1860 (31.99)	3.06 (2.68–3.51)	2.48 (1.98–3.12)
	Past antidepressant	332 (20.32)	872 (15.00)	2.65 (2.25–3.13)	1.96 (1.51–2.55)
Cancer	No antidepressant	445 (30.19)	2878 (53.11)	Reference	Reference
	Recent antidepressant	755 (51.22)	1791 (33.05)	3.11 (2.70–3.58)	3.25 (2.43–4.34)
	Past antidepressant	275 (18.66)	755 (13.93)	2.66 (2.23–3.19)	2.43 (1.70–3.48)
Respiratory	No antidepressant	201 (25.48)	1515 (54.79)	Reference	Reference
	Recent antidepressant	451 (57.16)	900 (32.55)	4.28 (3.49–5.25)	3.88 (2.72–5.54)
	Past antidepressant	137 (17.36)	393 (14.21)	2.82 (2.17–3.67)	1.76 (1.13–2.73)
All unnatural	No antidepressant	23 (16.79)	261 (52.41)	Reference	Reference
	Recent antidepressant	95 (69.34)	165 (33.13)	8.21 (4.73–14.24)	28.86 (6.60–126.15)
	Past antidepressant	19 (13.87)	72 (14.46)	4.11 (1.97–8.59)	0.98 (0.16–6.17)
Cumulative duration of antidepressant prescribing					
All-cause	No antidepressant	1460 (27.96)	1460 (27.96)	Reference	Reference
	1–6 months	1088 (20.83)	2378 (12.73)	3.39 (3.08–3.73)	3.10 (2.71–3.55)
	7–12 months	429 (8.22)	942 (5.04)	3.37 (2.95–3.85)	3.45 (2.86–4.16)
	13–23 months	514 (9.84)	1202 (6.44)	3.15 (2.79–3.55)	3.53 (2.97–4.19)
	≥24 months	1729 (33.11)	4260 (22.81)	3.15 (2.86–3.43)	3.40 (3.00–3.87)
Endocrine	No antidepressant	52 (25.12)	387 (52.02)	Reference	Reference
	1–6 months	42 (20.29)	85 (11.42)	3.98 (2.39–6.64)	3.10 (2.71–3.55)
	7–12 months	13 (6.28)	41 (5.51)	2.44 (1.21–4.94)	3.49 (2.87–4.17)
	13–23 months	14 (6.76)	55 (7.39)	2.16 (1.10–4.23)	3.53 (3.53–4.20)
	≥24 months	86 (41.55)	176 (23.66)	4.10 (2.68–6.27)	3.40 (3.00–3.87)
Cardiovascular	No antidepressant	483 (29.56)	3083 (53.03)	Reference	Reference
	1–6 months	315 (19.28)	792 (13.62)	2.72 (2.30–3.22)	2.59 (2.04–3.29)
	7–12 months	140 (8.57)	295 (5.07)	3.11 (2.47–3.92)	2.94 (2.07–4.16)
	13–23 months	161 (9.85)	353 (6.07)	2.96 (2.38–3.68)	3.15 (2.89–4.35)
	≥24 months	535 (32.74)	1291 (22.21)	3.01 (2.59–3.49)	2.87 (2.27–3.34)
Cancer	No antidepressant	445 (30.19)	2878 (53.11)	Reference	Reference
	1–6 months	359 (24.36)	669 (12.35)	3.88 (3.26–4.60)	4.07 (2.96–5.61)
	7–12 months	132 (8.96)	259 (4.78)	3.81 (2.98–4.87)	3.86 (2.47–6.03)
	13–23 months	128 (8.68)	360 (6.64)	2.58 (2.05–3.26)	3.18 (2.10–4.81)
	≥24 months	410 (27.82)	1253 (23.12)	2.42 (2.07–2.84)	3.00 (2.21–4.07)
Respiratory	No antidepressant	201 (25.48)	1515 (54.79)	Reference	Reference
	1–6 months	149 (18.88)	344 (12.44)	3.49 (2.70–4.51)	2.85 (1.95–4.16)
	7–12 months	69 (8.75)	138 (4.99)	2.47 (3.03–6.02)	4.53 (2.68–7.65)
	13–23 months	84 (10.65)	183 (6.62)	3.67 (2.70–5.00)	3.81 (2.38–6.11)
	≥24 months	286 (36.25)	627 (22.67)	4.01 (3.01–5.01)	4.67 (3.23–6.76)
All unnatural	No antidepressant	23 (16.79)	261 (52.41)	Reference	Reference
	1–6 months	22 (16.09)	47 (9.44)	6.61 (3.24–13.49)	7.71 (1.88–31.55)
	7–12 months	10 (7.30)	28 (5.62)	6.68 (2.71–16.51)	10.86 (2.48–47.63)
	13–23 months	20 (14.60)	31 (6.22)	11.28 (5.17–24.58)	23.73 (5.58–100.87)
	≥24 months	62 (45.26)	131 (26.31)	7.01 (3.92–12.53)	10.75 (3.44–33.61)
Number of different antidepressant agents prescribed					
All-cause	0	1460 (27.96)	1460 (27.96)	Reference	Reference
	1	2149 (41.15)	5295 (28.35)	3.13 (2.89–3.39)	3.64 (3.01–3.77)
	2	940 (18.00)	1990 (10.66)	3.73 (3.37–4.13)	3.71 (3.21–4.28)
	≥3	673 (12.89)	1228 (6.58)	4.52 (4.03–5.07)	4.93 (4.16–5.83)
Endocrine	0	52 (25.12)	387 (52.02)	Reference	Reference
	1	73 (35.27)	202 (27.15)	2.98 (1.97–4.53)	4.32 (2.15–8.65)
	2	46 (22.22)	88 (11.83)	4.62 (2.81–7.60)	3.79 (1.49–9.66)
	≥3	36 (17.39)	51 (6.85)	6.54 (3.78–11.32)	7.77 (2.92–20.69)
Cardiovascular	0	483 (29.56)	3083 (53.03)	Reference	Reference
	1	650 (39.78)	1697 (29.02)	2.73 (2.38–3.14)	2.75 (2.24–3.37)
	2	286 (17.50)	563 (9.68)	3.64 (3.03–4.36)	3.38 (2.59–4.40)
	≥3	216 (13.22)	383 (6.59)	4.23 (3.46–5.19)	4.30 (3.16–5.86)
Cancer	0	445 (30.19)	2878 (53.11)	Reference	Reference
	1	621 (42.13)	1536 (28.34)	3.05 (2.64–3.54)	3.88 (2.96–5.08)
	2	243 (16.49)	584 (10.78)	3.15 (2.60–3.81)	3.27 (2.30–4.63)
	≥3	166 (11.26)	343 (6.33)	3.76 (3.02–4.68)	5.93 (3.87–9.10)
Respiratory	0	201 (25.48)	1515 (54.79)	Reference	Reference
	1	342 (43.35)	773 (27.96)	3.78 (3.06–4.67)	3.83 (2.78–5.27)
	2	136 (17.24)	285 (10.31)	4.24 (3.23–5.56)	4.42 (2.92–6.68)
	≥3	110 (13.94)	192 (6.94)	5.42 (4.02–7.32)	5.73 (3.64–9.01)
All unnatural	0	23 (16.79)	261 (52.41)	Reference	Reference
	1	58 (11.65)	140 (28.11)	6.21 (3.48–11.06)	10.08 (3.41–29.85)
	2	33 (6.63)	58 (11.65)	8.80 (4.53–17.08)	16.88 (4.30–66.23)
	≥3	23 (16.79)	33 (6.63)	11.68 (5.50–24.79)	45.37 (7.92–259.89)

IRR, incidence rate ratio.

Individuals with comorbid depression and type 2 diabetes who were prescribed any antidepressant after starting oral antidiabetic medication, had considerably higher rates of all-cause mortality compared with those who were not prescribed an antidepressant (IRR 2.77, 95% CI 2.48–3.10).

In our two sensitivity analyses investigating the impact of missing data, we observed no evidence of a change in the effect estimates compared to the main analysis: we found an IRR of 2.72 (95% CI 2.36–3.14) when including only individuals with a completed value for ethnicity: we found an IRR of 2.77 (95% CI 2.48–3.09) when including only individuals with a completed value for body mass index.

We observed the highest IRR in unnatural causes of death (IRR 9.27, 95% CI 3.02–28.47), followed by endocrine causes (IRR 3.65, 95% CI 1.83–7.29), cancer (IRR 2.94, 95% CI 2.27–3.83), respiratory (IRR 2.94, 95% CI 2.13–4.04) and cardiovascular (IRR 2.27, 95% CI 1.86–2.77). The confidence intervals for unnatural causes of death were wide because of the small numbers of individuals experiencing these outcomes. The most common unnatural causes of death were suicide (*n* = 38) and falls (*n* = 38), followed by accidental poisoning (*n* = 18).

With regards to the timing of the antidepressant prescribing, we observed the most elevated rates of all-cause mortality in individuals who were recently prescribed an antidepressant (within 6 months of the outcome date) compared with those who were prescribed no antidepressant (IRR 3.19, 95% CI 2.82–3.61). In mortality from unnatural and endocrine causes, we observed elevated mortality rates in individuals who had recently been prescribed antidepressants, but not in those who only had antidepressant prescriptions >6 months in the past. We observed no evidence of a difference in the rates of mortality according to the timing of the antidepressant prescription for any of the other specific natural causes of death.

Rates of all-cause and cause-specific mortality did not vary according to the cumulative duration of antidepressant treatment.

For individuals with comorbid depression and type 2 diabetes, we observed increasingly higher mortality rates (for all-cause and every specific cause) as the amount of different antidepressant agents prescribed increased. Compared with no antidepressant prescriptions, the IRR for all-cause mortality in individuals who were prescribed one antidepressant agent was 3.64 (95% CI 3.01–3.77), the IRR for two antidepressant agents was 3.71 (95% CI 3.21–4.28) and the IRR for three antidepressant agents was 4.93 (95% CI 4.16–5.83).

## Discussion

Our study is the first to examine the association between antidepressant prescribing and cause-specific mortality in people with comorbid depression and type 2 diabetes. We found that people in this patient group had considerably higher rates of mortality when they were prescribed antidepressants, compared with those who were not prescribed antidepressants. This is the opposite to what we hypothesised – that treating depression with antidepressants would improve physical health through improving mental health, and so decrease mortality rates. Our findings are also the opposite to those from the study by Chen et al, who investigated the association between antidepressant prescribing and all-cause mortality.^11^ Chen et al did not adjust for comorbidities, whereas we adjusted for a wide range of comorbidities. However, this adjustment made little difference in our fully adjusted models, meaning that comorbidities that have been identified in primary care do not fully explain this disparity. The depression characteristics of the individuals included in our study may differ from those included by Chen et al, who included individuals with diagnosis of depression made by a psychiatrist, potentially representing individuals with more severe depression and accessing specialist care. Conversely, we included individuals with depression from a primary care population, where depression often does not receive a formal diagnosis, but may be based on the recording of symptoms. This may represent individuals with a broader range of depression severity.

National Institute for Health and Care Excellence guidelines only recommend that antidepressants are prescribed for people with physical comorbidities who are moderately to severely depressed.^18^ We were unable to adjust directly for depression severity because there is no routinely recorded variable for depression severity in our primary care data. Therefore, people in our study who were prescribed antidepressants may have been more severely depressed than those who were not prescribed antidepressants. Furthermore, the prescription of an antidepressant does not necessarily indicate successfully treated depression. An individual may not take the antidepressants prescribed, or they may not respond to treatment. As such, the increased rates of mortality in our study may be attributable to depression, rather than antidepressant treatment (confounding by indication). This explanation is supported by the fact that we observed no difference in the rates of mortality according to increased exposure to antidepressant treatment, measured by the cumulative duration of treatment. Individuals who are prescribed antidepressants for longer durations, conceptually, may be more likely adhere to treatment. Again, the fact that this potential increased exposure to antidepressants through increased adherence had no effect, suggests that the increased rates of mortality in our study may be more attributable to depression, rather than antidepressant treatment itself. We did, however, observe a further increase in rates of mortality for individuals who were prescribed more different antidepressant agents, potentially representing ‘complex-to-treat’ depression.

We observed the highest increase in mortality in individuals who died from unnatural causes (including suicide). This was only observed in those who were recently prescribed antidepressants, suggesting an acute association. Some common antidepressants may worsen suicidal ideation during the initial phases of treatment and after recently stopping because of side-effects or withdrawal symptoms of agitation and activation.^19^ However, in randomised controlled trials (RCTs) in the general population, antidepressants have been shown to reduce the risk of suicide.^20^ It may be that, in our study, antidepressants simply indicate current depression (which may not be successfully treated). Although all individuals that we included during the study had a clinical code for depression at some point during the observed period, these codes could have been years before the date of death. Therefore, people who were not prescribed antidepressants may not have been depressed at the same point in time as those who were prescribed antidepressants. It should be noted, however, that some unnatural causes of mortality that are not related to suicide, such as falls and accidental poisoning, may also be caused by antidepressant side-effects or overdose.^21^ With the available data, it is not possible to discern whether the association with unnatural causes of mortality can attributed to antidepressant drugs themselves, or to current (and unsuccessfully treated) depression.

We also only observed increased rates of endocrine mortality when antidepressants had been prescribed recently. Acute, fatal endocrine events may include hyperglycaemic or hypoglycaemic crises. We are aware of one large (*N* = 133 599) population-based study in Taiwan, by Lee et al, which found that individuals with comorbid depression and diabetes (type unspecified) were 64% less likely to experience hyperglycaemic crises when they had been prescribed antidepressants, compared with those who had not.^22^ However, Lee et al only included individuals with more severe depression, whereas we were unable to account for depression severity, which may have confounded our findings. Depression is known to increase the risk of both of hyperglycaemia and hypoglycaemia.^23^

In the long term, rates of mortality were similarly elevated across the three major causes of death – cardiovascular, cancer and respiratory – in individuals who were prescribed antidepressants compared with those who were not. If antidepressant prescribing was associated specifically with a decline in diabetic health, we would have expected to see the most elevated rates of mortality in cardiovascular causes of mortality, as type 2 diabetes is a major risk factor for this and it this is the leading cause of death in this patient group. As this was not the case, antidepressant prescribing may be a marker for worse overall health generally, rather than specifically diabetic health. We propose, however, that it is unlikely that the increased risk of mortality from these different causes is directly caused by antidepressant drugs, as the risk was equally elevated across cardiovascular, cancer and respiratory causes, which are caused by different mechanisms. Therefore, there is no theoretical explanation to support a direct effect of antidepressants. The exception to this is that all conditions are linked to smoking;^24^ however, we adjusted for smoking status in our analysis and this had very little effect.

### Strengths and limitations

With a sample size of 23 897, this study is over 70 times larger than all studies combined in the Cochrane meta-analysis of RCTs investigating antidepressant treatment in this patient group.^7^ The median duration of observation in our study was 7 years, which is usually not feasible for RCTs. In addition, because of strict inclusion criteria, RCTs may not be generalisable to the population of interest. All RCTs included in the Cochrane review excluded individuals with the most severe depression, and the majority excluded individuals with common comorbidities and co-prescriptions.^7^ As this was a nested case–control study using incident density sampling from a cohort of all individuals registered at CPRD general practices who had depression and had started treatment for type 2 diabetes, this minimised the selection bias that occurs in classic case–control studies.^25^ However, as we matched controls to cases who had died based on age, the median age of people included in this study was 69 years. As such, the findings of this study may only be generalisable to older adults.

Antidepressant prescribing decisions will be made based on an indication of requiring treatment, clinician prescribing habits, patient preferences and local pressures in access to care. These reasons may themselves introduce bias, if they also offer alternative explanations for increased rates of mortality. We attempted to balance a wide range of confounders by individual-level matching and adjusting for confounders in our model. However, we could not adjust for depression severity itself. There is no consistently recorded measure of depression severity in UK primary care data. Further research is required to investigate whether there may be suitable markers of depression severity that predict antidepressant treatment in UK primary care data.

All confounders included in this study were measured at study entry, whereas the median duration that patients were observed for was 7 years. Individuals who were prescribed antidepressants may have seen a decline in physical and mental health since study entry, potentially because of factors that were not available from EHR data. This could include person-level socioeconomic status, lifestyle, life events, social factors, cultural factors and environmental factors.^26^ In addition, unless the onset of fatal disease occurred before the study entry date, it was not possible to see whether this occurred before or after antidepressant prescribing. Therefore, our findings may have been a result of reverse causation, whereby the distress from physical conditions may have caused episodes of depression requiring antidepressant treatment. However, because of our case–control study design, we were able to examine different patterns of antidepressant exposure: the timing of the antidepressant prescribing, the cumulative duration and being prescribed a higher number of different antidepressant agents (potentially indicating treatment failure). This allowed us to strengthen our causal understanding of the association between antidepressant prescribing and different causes of mortality in individuals with comorbid depression and type 2 diabetes.

Because of smaller sample sizes, we did not investigate differences between different antidepressant drug classes, which have different efficacies and side-effect profiles. Further research is required in this area.

### Conclusions and implications

Our study has identified antidepressant prescribing as a marker for considerably higher rates of mortality in people with comorbid depression and type 2 diabetes in UK primary care. There is not sufficient evidence to suggest that antidepressant drugs themselves are causing the increased rates of mortality. Conversely, we propose that individuals in UK primary care who are prescribed antidepressants have unmeasured characteristics, such as more severe depression or adverse socioeconomic factors, that lead to higher rates of mortality from a range of causes. Although these individuals are being treated with antidepressant medication, this does not appear to sufficiently improve depressive symptoms such that the negative effects of depression on physical health are negated. Thus, unsuccessfully treated depression may be of particular concern in this patient group. Individuals with comorbid depression and type 2 diabetes who are being treated with antidepressants should be closely monitored and offered enhanced holistic care to improve their physical and mental health. Further research is required to understand the causal pathway between antidepressant prescribing and increased mortality rates in this patient group.

## Supporting information

Jeffery et al. supplementary material Jeffery et al. supplementary material

## Data Availability

Data are available from the CPRD following study-specific protocol approval via CPRD's Research Data Governance Process.

## References

[1] Vos T, Allen C, Arora M, Barber RM, Brown A, Carter A, et al. Global, regional, and national incidence, prevalence, and years lived with disability for 310 diseases and injuries, 1990–2015: a systematic analysis for the global burden of disease study 2015. Lancet 2016; 388(10053): 1545–602. 27733282 10.1016/S0140-6736(16)31678-6 PMC5055577

[2] Khaledi M, Haghighatdoost F, Feizi A, Aminorroaya A. The prevalence of comorbid depression in patients with type 2 diabetes: an updated systematic review and meta-analysis on huge number of observational studies. Acta Diabetol 2019; 56(6): 631–50. 30903433 10.1007/s00592-019-01295-9

[3] Lustman PJ, Anderson RJ, Freedland KE, De Groot M, Carney RM, Clouse RE. Depression and poor glycemic control: a meta-analytic review of the literature. Diabetes Care 2000; 23(7): 934–42. 10895843 10.2337/diacare.23.7.934

[4] Gonzalez JS, Peyrot M, McCarl LA, Collins EM, Serpa L, Mimiaga MJ, et al. Depression and diabetes treatment nonadherence: a meta-analysis. Diabetes Care 2008; 31(12): 2398–403. 19033420 10.2337/dc08-1341 PMC2584202

[5] de Groot M, Anderson R, Freedland KE, Clouse RE, Lustman PJ. Association of depression and diabetes complications: a meta-analysis. Psychosom Med 2001; 63(4): 619–30. 11485116 10.1097/00006842-200107000-00015

[6] van Dooren FEP, Nefs G, Schram MT, Verhey FRJ, Denollet J, Pouwer F. Depression and risk of mortality in people with diabetes mellitus: a systematic review and meta-analysis. PLoS One 2013; 8(3): e57058. 23472075 10.1371/journal.pone.0057058 PMC3589463

[7] Baumeister H, Hutter N, Bengel J. Psychological and pharmacological interventions for depression in patients with coronary artery disease. Cochrane Database Syst Rev 2011; 9: CD008012. 10.1002/14651858.CD008012.pub3 PMC7389312 21901717

[8] British National Formulary. *Sertraline*. National Institute for Health and Care Excellence, 2022 (https://bnf.nice.org.uk/drug/sertraline.html).

[9] British National Formulary. *Mirtazapine*. National Institute for Health and Care Excellence, 2022 (https://bnf.nice.org.uk/drug/mirtazapine.html).

[10] Jeffery A, Buckman JEJ, Francis E, Walters K, Wong ICK, Osborn D, et al. A systematic review of long-term antidepressant outcomes in comorbid depression and type 2 diabetes. medRxiv [Preprint] 2022. Available from: https://www.medrxiv.org/content/10.1101/2022.04.11.22273519v1 [cited 3 May 2022].

[11] Chen HM, Yang YH, Chen KJ, Lee Y, McIntyre RS, Lu ML, et al. Antidepressants reduced risk of mortality in patients with diabetes mellitus: a population-based cohort study in Taiwan. J Clin Endocrinol Metab 2019; 104(10): 4619–25. 31265070 10.1210/jc.2018-02362

[12] Doos L, Roberts EO, Corp N, Kadam UT. Multi-drug therapy in chronic condition multimorbidity: a systematic review. Fam Pract 2014; 31(6): 654. 25192902 10.1093/fampra/cmu056 PMC5942538

[13] Medicines and Healthcare products Regulatory Agency. *Clinical Practice Research Datalink (CPRD)*. Medicines and Healthcare products Regulatory Agency, 2024 (https://www.cprd.com/). 10.1136/bmj.330.7497.917 PMC556324 15845955

[14] Herrett E, Gallagher AM, Bhaskaran K, Forbes H, Mathur R, van Staa T, et al. Data resource profile: clinical practice research datalink (CPRD). Int J Epidemiol 2015; 44(3): 827–36. 26050254 10.1093/ije/dyv098 PMC4521131

[15] Wolf A, Dedman D, Campbell J, Booth H, Lunn D, Chapman J, et al. Data resource profile: clinical practice research datalink (CPRD) Aurum. Int J Epidemiol 2019; 48(6): 1740. 30859197 10.1093/ije/dyz034 PMC6929522

[16] British National Formulary. *Metformin Hydrochloride.* National Institute for Health and Care Excellence, 2022 (https://bnf.nice.org.uk/drug/metformin-hydrochloride.html).

[17] Essebag V, Platt RW, Abrahamowicz M, Pilote L. Comparison of nested case-control and survival analysis methodologies for analysis of time-dependent exposure. BMC Med Res Methodol 2005; 5: 5. 10.1186/1471-2288-5-5 PMC548149 15670334

[18] National Institute for Health and Care Excellence (NICE). Depression in Adults: Recognition and Management. Clinical Guidance [CG90]. NICE, 2009 (https://www.nice.org.uk/guidance/cg90). 31990491

[19] Coupland C, Hill T, Morriss R, Arthur A, Moore M, Hippisley-Cox J. Antidepressant use and risk of suicide and attempted suicide or self harm in people aged 20 to 64: cohort study using a primary care database. BMJ 2015; 350: h517. 10.1136/bmj.h517 PMC4353276 25693810

[20] Cipriani A, Barbui C, Geddes JR. Suicide, depression, and antidepressants. BMJ 2005; 330(7488): 373–4. 15718515 10.1136/bmj.330.7488.373 PMC549094

[21] Rockett IR, Kapusta ND and Bhandari R. Suicide misclassification in an international context: revisitation and update. Suicidology Online 2011; 2: 48–61.

[22] Lee HM, Yang YC, Chen SF, Hsu CY, Shen YC. Risk of hyperglycemic crisis episode in diabetic patients with depression: a nationwide population-based cohort study. J Diabetes Complications 2020; 34(3): 107509. 10.1016/j.jdiacomp.2019.107509 31864898

[23] Jung A, Du Y, Nübel J, Busch MA, Heidemann C, Scheidt-Nave C, et al. Are depressive symptoms associated with quality of care in diabetes? Findings from a nationwide population-based study. BMJ Open Diabetes Res Care 2021; 9(1): e001804. 10.1136/bmjdrc-2020-001804 PMC7986897 33753346

[24] NHS England. *What are the Health Risks of Smoking?* NHS England, 2022 (https://www.nhs.uk/common-health-questions/lifestyle/what-are-the-health-risks-of-smoking/).

[25] Sedgwick P. Nested case-control studies. BMJ 2010; 340: c2582. 20484347 10.1136/bmj.c2582

[26] Dykxhoorn J, Fischer L, Bayliss B, Brayne C, Crosby L, Galvin B, et al. Conceptualising public mental health: development of a conceptual framework for public mental health. BMC Public Health 2022; 22(1): 1–15. 35870910 10.1186/s12889-022-13775-9 PMC9308351

