# Supplementary material for: Antidepressant treatment and mortality in people with comorbid depression and type 2 diabetes: UK electronic health record study
Source: BJPsych Open. 2024 Apr 12;10(3):e79. doi: 10.1192/bjo.2024.33 (PMC11060070; doi:10.1192/bjo.2024.33)
Supplement: Jeffery et al. supplementary material [file S2056472424000334sup001.docx]

**Supplementary box 1 – antidepressants drugs included**

SSRIs: Citalopram, escitalopram, fluoxetine, fluvoxamine, paroxetine, sertraline;

SNRIs: Duloxetine, venlafaxine;

TCAs: Amitriptyline >= 50mg per day*, amoxapine, clomipramine, dosulepin, oral doxepin, imipramine, lofepramine, maprotiline, nortriptyline >=50mg per day*, trimipramine;

MAOIs: Isocarboxazid, phenelzine, tranylcypromine;

Atypical: Agomelatine, mianserin, mirtazapine, moclobemide, nefazodone, reboxetine, trazodone, tryptophan, vortioxetine.

*Amitriptyline and nortriptyline were included only at the anti-depressant dose of >= 50mg per day, rather than lower hypnotic or neuralgia doses.

**Supplementary box 2 – full details of confounding variables included in our model**

Demographic characteristics: In addition to the variables used for matching, we also included ethnicity. Where ethnicity was missing, we recoded this as “White”– as the CPRD population has been found to be representative of the UK population in terms of ethnicity (1,2), 93% of more individuals with missing ethnicity would be expected to be of White ethnicity. This approach is in line with other research studies using the CPRD (3).

Health characteristics: We included a range of health characteristics that may be confounders through an association with antidepressant use (either directly or indirectly through an association with depression) and mortality (either directly or indirectly through an association with poor overall physical health, or health behaviours):

The following comorbidities (as individual comorbidities) based on codes from the Elixhauser comorbidity code list (4): alcohol abuse, blood loss anaemia, cardiac arrythmia, chronic pulmonary disease, coagulopathy, deficiency anaemia (iron/B12), drug abuse, fluid and electrolyte disorders, hypertension (uncomplicated), hypertension with end organ damage, hypothyroidism, liver disease, lymphoma (history), metastatic cancer, other neurological disorders, paralysis, peptic ulcer disease, peripheral vascular disease, psychoses, pulmonary circulation disorders, renal disease, rheumatoid arthritis and collagen diseases, solid tumor or leukaemia, valvular disease;

The most recent recorded BMI value categorised into “normal” (<25 kg/m2), “overweight” (25 to <30 kg/m2), and “obese” (30+ kg/m2); where no value for BMI was available, we estimated the value using multiple imputation;

Smoking status categorised into “current smoker” (most recent smoking code related to current smoking within 12 months before study entry), “ex-smoker” (most recent smoking code related to historic smoking, or most recent code related to current smoking more than 12 months before study entry, or most recent code related to non-smoking with prior codes related to current or historic smoking), “non-smoker” (no codes related to smoking, or non-smoking codes only);

Type 2 diabetes duration, defined as the number of months between the first diabetes related code (this could include diagnosis, symptom, process of care, or medication) and the date of the first oral antidiabetic medication prescription;

Number of primary care contacts recorded in the 12 months prior to the study entry date – this included any face-to-face contact or phone calls. Higher numbers of primary care contacts were intended to represent worse overall physical and/or mental health.

Medication history: We included the number of different pharmacological medications prescribed (excluding vaccinations, topical medications and supplements) in the 90 days before the study entry. It was not possible to account for markers of depression and depression severity at study entry. Therefore, to indicate previous depression at a severity requiring pharmacological treatment, we included any previous antidepressant prescription in the 12 months prior to study entry.

We did not include glycaemic control as a potential confounder, given the study inclusion criteria of starting oral antidiabetic medication (study entry) meant that all participants would be expected to have uncontrolled blood sugar levels at the time of study entry.

1. Herrett E, Gallagher AM, Bhaskaran K, Forbes H, Mathur R, Staa T van, et al. Data Resource Profile: Clinical Practice Research Datalink (CPRD). Int J Epidemiol [Internet]. 2015 Jun 1 [cited 2022 Jan 13];44(3):827–36. Available from: https://pubmed.ncbi.nlm.nih.gov/26050254/

2. Wolf A, Dedman D, Campbell J, Booth H, Lunn D, Chapman J, et al. Data resource profile: Clinical Practice Research Datalink (CPRD) Aurum. Int J Epidemiol. 2019 Dec 1;48(6):1740-1740G.

3. Hippisley-Cox J, Coupland C, Vinogradova Y, Robson J, Minhas R, Sheikh A, et al. Predicting cardiovascular risk in England and Wales: prospective derivation and validation of QRISK2. BMJ [Internet]. 2008 Jun 28 [cited 2022 Jan 13];336(7659):1475–82. Available from: https://pubmed.ncbi.nlm.nih.gov/18573856/

4. Elixhauser A, Steiner C, Harris DR, Coffey RM. Comorbidity measures for use with administrative data. Med Care [Internet]. 1998 [cited 2022 Apr 12];36(1):8–27. Available from: https://pubmed.ncbi.nlm.nih.gov/9431328/
